# Living arrangements and property ownership among families in India

**DOI:** 10.3389/fsoc.2026.1865674

**Published:** 2026-08-25

**Authors:** Shweta Kalla Baxi, Sarthak Gaurav, Usha Ananthakumar

**Affiliations:** Shailesh J. Mehta School of Management, Indian Institute of Technology Bombay, Mumbai, India

**Keywords:** family, India, living arrangement, property ownership, strategic exchange

## Abstract

**Introduction:**

Family is an important source of intergenerational support for older adults in Indian society due to strong norms of filial obligation embedded within the kinship system. However, as urbanization and migration affect the feasibility of traditional intergenerational support arrangements, incentive-based mechanisms may play an important role in shaping intergenerational support. Against this backdrop, this paper examines whether residential proximity to parents is associated with ownership of parental household assets.

**Methods:**

We use data from the Longitudinal Ageing Study in India (LASI) and a conditional fixed-effects logit model to compare siblings within the same family with respect to proximity to their parents and their ownership of parental household property.

**Results:**

We find that adult children co-residing with their parents are more likely than their siblings residing elsewhere to be reported as owners of parental household property. The association is stronger among wealthier households and older parents.

**Discussion:**

These findings are consistent with an exchange-based interpretation of intergenerational support and suggest that such considerations may coexist with strong norms of informal support.

**JEL Codes:**

D64, J14, G51

## 1 Introduction

Family has traditionally served, and continues to serve as the primary institution providing security for older adults in India. Unlike other countries where formal welfare systems are well developed and critical for late-life support, older adults in India largely rely on family members for economic, emotional, and caregiving assistance. This support is commonly provided through intergenerational co-residence, wherein aged parents live together with their adult children in the same household (Karve, 1968; Sathyanarayana et al., 2014; Uberoi, 1994). Cultural and social norms have significantly contributed to the persistence of these intergenerational living arrangements (Kumar, 1995), which have their roots in the early Indian literary texts that prescribed parental reverence and servitude (Dyson and Moore, 1983; Olivelle, 2004).

Alongside norms, altruism and reciprocity also play an implicit role in this intergenerational exchange. The altruistic perspective posits that intergenerational exchanges are need-based as parents help resource-constrained children, and children help older parents facing health, financial, or other challenges (Kunemund et al., 2005). The altruistic behavior of children toward caring for their elderly parents are widely documented in the family and aging literature in India (Karve, 1968; Uberoi, 1994; Ugargol and Bailey, 2018). Alternatively, the exchange theory suggests that children provide support to aging parents in exchange for parental transfers made either during the parents' lifetimes or through bequests (Bernheim et al., 1985; Cox and Rank, 1992). Research has also shown that adult children may devote greater time and effort to their parents when parental financial transfers are involved, although most of this evidence comes from contexts outside India (Brown, 2006; Fu, 2019; Rao et al., 2025). Because residential proximity is an important dimension of intergenerational support, families' living arrangements have also been shown to emerge from the interplay of filial obligations, altruistic behavior, and reciprocal exchanges (Fu, 2019; Wang et al., 2019).

In India, the intergenerational co-residence of parents with married children, particularly sons, remains the dominant living arrangement (Rajan and Kumar, 2025; Sathyanarayana et al., 2014). However, modernization, industrialization, urbanization, and increased migration have made it more difficult for families to maintain these traditional living arrangements, even as norms of filial support remain strong (Cohen, 2000; NSO, 2021). These changes are particularly important in India, where rapid population aging coincides with low pension penetration, leaving family support as the primary source of old-age security for many households. Demographically, the country's age structure is changing due to lower mortality and reduced fertility, leading to an increase in the number of seniors (NSO, 2021). The formal old-age support system, however, is still in a nascent stage, and a large fraction of the population is not covered by pensions (OECD, 2023). Even as norms and moral obligations persist, reciprocal exchange may assume greater importance in forming intergenerational support arrangements. If parents use property ownership as part of such arrangements, children who are better positioned to provide support, such as co-resident children, may be more likely to be allocated property. This raises an important question we address in this paper: are adult children who live with or in close proximity to their parents more likely than their siblings to be reported as owners of parental household property?^1^

India provides a particularly relevant setting for examining this relationship. In the exchange literature, reciprocity is often studied through financial transfers and bequests from parents. However, the composition of household wealth in India differs markedly from that of many developed countries (Ramadorai, 2017). In India, land and buildings account for nearly 90% of household assets, making wealth predominantly immovable in nature (MOSPI, 2019). At the same time, limited financial assets are largely absorbed by parental obligations to fund children's education and marriage, limiting the scope for transfers later in life (Anukriti et al., 2022; Bloch et al., 2004; Kaleem and Akhtar, 2025). Consequently, property ownership may offer a useful lens through which to examine intergenerational arrangements within families. While a large literature has examined family support and living arrangements in India, relatively little is known about whether differences in adult children's living arrangements are associated with ownership of parental household immovable assets. If such an association exists, it may reflect patterns that are consistent with a strategic-exchange interpretation, although alternative explanations are also possible.

Our study is among the first to examine the relationship between ownership of parental household property and adult children's proximity to parents in India. In doing so, the paper complements existing research on inter-vivos transfers (Chou, 2008; Ciani and Deiana, 2018; Cunningham et al., 2013; Ho, 2019; Rao et al., 2025) and end-of-life bequests (Brown, 2006; Groneck, 2017; Hamaaki et al., 2019). The study also offers contextual novelty by offering insights from India, where strong norms of filial piety coexist with a distinctive asset portfolio, rapid population aging, and evolving family dynamics. Taken together, these contributions advance our understanding of the role that property ownership may play within families in asset-heavy but liquidity-constrained contexts.

## 2 Background literature and hypothesis development

The Indian family system has historically been organized around a joint living arrangement in which multiple generations co-reside within the same household (D'cruz and Bharat, 2001; Karve, 1968; Uberoi, 1994). This structure remains prevalent to this date, with more than 80% of older adults in India living with their children or extended kin (Sathyanarayana et al., 2014). The joint household exemplifies the structural dimension of intergenerational solidarity, and provides the foundation for other dimensions, such as associational, affectual, consensual, functional, and normative, to exist (Bengtson and Roberts, 1991). Parents typically support younger generations through expenditures on consumption, education, and marriage (Becker, 1991). Similarly, older parents and grandparents receive care and assistance from adult children, grandchildren, or other household members (Jothikaran et al., 2023).

Therefore, intergenerational relationships in India are embedded within the larger kinship system, which prescribes mutual interdependence among all household members (D'cruz and Bharat, 2001; Kumar, 1995). Supporting older parents and other older kin is not just a private arrangement between individuals but a moral and social responsibility shared by all members (Karve, 1968). Parents are accorded a higher status within the family, as also reflected in the fact that they are more often than not considered the household head (Sathyanarayana et al., 2014). There do exist heterogeneities in family and kinship arrangements with respect to region and caste affiliation, as a result of differences in marriage systems and social organization (Karve, 1968; Uberoi, 1994). For instance, the traditional household arrangements have a North-South divide, with stronger adherence in the Northern regions (Dyson and Moore, 1983). Notwithstanding the heterogeneity, family remains a dominant form of informal social security even in contemporary society.

However, the joint family system has become difficult to sustain in recent decades. Multiple demographic and socioeconomic factors have reshaped family living patterns and, consequently, affected the support that elderly members could easily access earlier (Chakravorty et al., 2021). In India, like much of Asia, rapid urbanization and internal migration have increased spatial separation between parents and their adult children, as younger cohorts move to cities or other states in search of education and employment (Bloom et al., 2010; Zhao and Shangguan, 2025). Added to this is a growing preference for nuclear living arrangements that emphasize autonomy and privacy over collective interdependence (*ibid*.). Declining fertility rates are also a contributory factor, as there are fewer children to co-reside with ((IIPS & ICF, 2022). The proportion of elderly individuals living alone or only with a spouse has risen slowly, but gradually (Sathyanarayana et al., 2014).

The consequences of these shifts are especially pronounced for current older adults. Life expectancy in India has increased markedly from around 50 years in 1970-75 to approximately 70 years in 2016-20, implying that older individuals now face longer post-retirement periods for which they must remain financially secure (GOI, 2022). At the same time, expectations of financial and physical support from children are strong (Allendorf, 2020), while alternative sources of old-age security remain limited.

Formal measures of social protection in the country, which have the potential to displace informal support (Wang et al., 2025), are still in their early stages. State support in the country is limited and pension coverage remains low (OECD, 2023). Additionally, almost 90% of the workforce in India is in the unorganized sector, where access to social insurance is limited (Mehrotra, 2019; Sheikh and Gaurav, 2020). Together, these factors imply that the state plays a limited role in providing old-age income security.

Further compounding the issue are the patterns of asset ownership in the country. Household wealth is heavily concentrated in non-liquid assets, especially real estate (MOSPI, 2019). The market for reverse mortgages, which could have offered some flexible options to homeowners to convert their housing wealth into income in older age, is still very nascent in the country (Ramadorai, 2017). Even in urban areas with better access to financial institutions (Lenka and Barik, 2018), nearly 87 percent of wealth is held in immovable property (MOSPI, 2019). Thus, much of the wealth is locked with limited liquidity and possibly preserved for intergenerational transfer rather than personal security in later life (Ramadorai, 2017). In this context, household immovable assets become particularly relevant for understanding intergenerational support arrangements.

Although filial norms remain important for old-age support, research suggests that intergenerational support reflects the coexistence of normative obligations, altruistic motivations, and reciprocal exchange (Alessie et al., 2014; Silverstein and Giarrusso, 2010). These mechanisms are not mutually exclusive and may operate alongside one another within the same family relationships. However, demographic and socioeconomic changes may increase the relevance of reciprocal exchange in intergenerational support arrangements. Therefore, we draw on strategic exchange theory as a possible lens through which to interpret the association between adult children's residential proximity to parents and ownership of parental household immovable assets.

Exchange theory provides a useful framework for understanding reciprocal behavior, which suggests that parents may use transfers to encourage support from children (Bernheim et al., 1985; Cox, 1987; Cox and Rank, 1992). Such transfers typically take the form of inter vivos^2^ financial transfers or bequests of property or other non-financial assets. However, as household wealth in India is largely concentrated in residential and land assets, property may represent an important dimension through which intergenerational arrangements are structured. While previous studies have primarily examined exchange of financial transfers and bequests in other countries, less attention has been paid to whether ownership of parental immovable assets is associated with children's residential proximity to parents. Hence, existing studies may be limited in explaining the nature of these arrangements in the Indian context.

The relationship between property ownership and intergenerational support is also shaped by gender in India. Despite legal reforms, sons are more likely than daughters to inherit family property and co-reside with older parents under patrilocal and patrilineal structures in most parts of the country (Deininger et al., 2013). Consequently, the relationship between co-residence, caregiving, and property ownership may be embedded within broader gendered norms. Furthermore, such an arrangement may be particularly salient in families with multiple children, wherein parents can differentiate among children (Bernheim et al., 1985) Although direct transfer decisions are not observed in our data, patterns of property ownership offer a useful perspective on how resources are allocated within families.

Following exchange theory, parents may allocate resources in ways that encourage support from their adult children. Since co-resident children are often better positioned to support aging parents (Fu, 2019), they occupy a particularly important position in intergenerational support arrangements. It is well established that parent-child co-residence may arise for a variety of reasons, including economic dependency of the child on the parent, marriage, housing and space constraints, employment and migration choices, family relationships, social norms, and individual preferences (Croll, 2006; Esteve and Reher, 2021; Karve, 1968; Mishra and Kaur, 2021; Ugargol et al., 2016). Nevertheless, co-residence is regarded as an important indicator of support in aging literature in India and in other countries (Bengtson and Roberts, 1991; Chappell, 1991; DaVanzo and Chan, 1994; Jothikaran et al., 2023; Sathyanarayana et al., 2014). For older parents who may face declining health and who might have a greater need for care, co-residence with children can be particular helpful (Ugargol et al., 2016). Therefore, if property ownership forms part of such reciprocal arrangement, ownership may be more prevalent among co-resident children than among their siblings who reside elsewhere. Note, however, that given social norms, parents may prefer not to differentiate among their children in the division of assets (Bernheim and Severinov, 2003). Cultural expectations of fairness and family harmony may, hence, lead to an equal division of assets, irrespective of co-residence or caregiving. Accordingly, our first hypothesis states:

**Hypothesis 1**: *Co-resident children in India are more likely to be owners of the parental household's immovable assets than their siblings who do not co-reside*.

Exchange theory further suggests that support provision may help explain the association between residential proximity and resource allocation. Co-resident children face lower marginal costs of caregiving due to shared living arrangements and socially embedded expectations of support (Fu, 2019). Consistent with this premise, evidence from the Longitudinal Ageing Study in India (LASI) shows that more than 90 percent of caregiving is provided by individuals residing in the same household as the care recipient. This pattern suggests that caregiving may help explain the association between co-residence and property ownership. We therefore examine whether the observed association between co-residence and ownership is reduced after accounting for caregiving provision. Hence, our second hypothesis is as follows.

**Hypothesis 2**: *The association between co-residence and property ownership is attenuated after accounting for caregiving provision*.

In the analyses that follow, we employ data from the Longitudinal Ageing Study in India (LASI) to test these hypotheses.

## 3 Data and methods

### 3.1 Data

We use the first wave of the data from the Harmonized Longitudinal Ageing Study in India (LASI) (Chien et al., 2023; IIPS, 2020). LASI, the world's largest national study on health and retirement (Bloom et al., 2021), covered nearly 72,000 individuals from more than 43,000 households across rural and urban India and gathered comprehensive information on the economic, social, psychological, and health conditions of older adults. Since subsequent waves of LASI are not yet available, we use the baseline survey conducted in 2017–18; accordingly, our analysis is based on cross-sectional data.

We begin by retaining households in which either the household head was interviewed or, if the head was not interviewed, the head's spouse was interviewed. We also limit respondents to those aged 45 years or older, consistent with the LASI design and a stage of life at which intergenerational arrangements become more salient (Cagetti, 2003). We further restrict the sample to households with at least one living adult child and complete information on household immovable assets and all study variables, yielding an analytic sample of 33,198 household heads with 105,094 adult children. Our primary analysis employs a conditional fixed-effects logit model to identify associations using within-family variation in ownership of the parental household's immovable property among adult children of the household head. Consequently, only those families are included in the estimation in which at least one adult child owns the parental household's immovable property, while at least one other adult child does not. Families in which all adult children either own or do not own the parental household's immovable property are excluded because they provide no within-family variation in ownership. By the same logic, households with only one adult child are also excluded because the model compares property ownership across siblings, and no such comparison is possible when there is only one child. Thus, our final estimation sample comprises 9,124 adult children from 2,365 parental households.^3^ Table 1 presents the sample selection process. Appendix Table A1 compares the characteristics of the initial analytic sample and the final estimation sample.

**Table 1:** Construction of the final estimation sample.

Step	Sample restriction	Households	Children	Excluded households
1	Harmonized LASI Wave 1 households	43,364	-	-
2	Household head or spouse interviewed	38,641	-	4,723
3	Respondent aged ≥45 years	37,646	-	995
4	Information available on household immovable assets	36,973	-	673
5	At least one living adult child	33,281	1,06,402	3,692
6	Complete information on all study variables (analytic sample)	**33,198**	**1,05,094**	83
7	Final estimation sample for the conditional fixed-effects logit	**2,365**	**9,124**	30,833

The analytic sample includes all households that meet the study eligibility criteria and have complete information on the variables used in the analysis. The final estimation sample is smaller because conditional fixed-effects logit models are estimated using only households that contribute to the conditional likelihood. Consequently, households with no within-family variation in the dependent variable are excluded automatically from estimation. The values in bold indicate the final analytic and estimation sample used for the study.

### 3.2 Measures

#### 3.2.1 Asset ownership

Our main dependent variable is a binary indicator of whether a child owns any of the immovable assets reported within the parental household's asset portfolio, including housing, non-housing property, cultivable land, or non-cultivable land. The analysis is therefore limited to ownership of assets reported within the parental household.

For each asset, LASI records whether the owner is a household member or a non-household member. When the owner is a household member, the specific household member(s) are identified. For the current residence (of the household head), LASI additionally identifies whether a non-household owner is a non-household child, and we incorporate this information in constructing the ownership variable. However, for non-household owners whose relationship to the household head is not identified, we attribute ownership to non-resident children.^4^ We acknowledge that this assumption may not always hold, particularly when the head has multiple non-resident children or when the owner is a non-household relative. If some non-household owners are relatives other than adult children, this coding may overstate ownership among non-resident children. Such non-differential misclassification would likely attenuate the estimated association by classifying some true non-household relative owners as non-resident children, thereby reducing the observed difference in ownership between co-resident and non-resident children.

We assess the sensitivity of this coding assumption using two robustness analyses, both based on ownership of the current residence as the dependent variable. First, we estimate the model by classifying non-resident children as owners only when they are explicitly identified as non-household child owners. Second, we further restrict the analysis to households with only one non-resident child, thereby eliminating ambiguity in assigning ownership to a specific child. These analyses assess the sensitivity of our findings to the ownership coding used in the primary specification.

Finally, ownership may reflect an adult child's own financial contribution rather than an intergenerational transfer. To examine this possibility, we estimate an additional robustness specification using only housing assets acquired before the child reached adulthood (18 years). Because information on the year of acquisition is available only for the current residence (of the household head), this analysis is limited to ownership of the current residence. Restricting the sample in this way reduces the likelihood that the child financed the original acquisition of the property through their own earnings. However, it does not establish that the child became an owner before reaching adulthood, as ownership could have been transferred or shared after the property was acquired. Accordingly, this robustness check addresses concerns about financing the original purchase but does not resolve uncertainty about the timing of the ownership transfer, which we cannot address with the existing data.

#### 3.2.2 Intergenerational support

We measure the proximity of the child to the parents using three levels in order of their distance, distinguishing between 0 (co-residing with parent), 1 (not co-residing with parent but living within the same city or village), and 2 (not co-residing with parent and living outside the city or village). The definition captures the dynamics of household choice, as living within the city but outside the parental household might reflect the child exercising some choice in living arrangements. We acknowledge that co-residence alone may not indicate that the child will provide support. However, proximity reduces the cost of care provision and hence is regarded as an indicator of potential caregiving following the wider literature (Bengtson and Roberts, 1991; Chappell, 1991; DaVanzo and Chan, 1994).

Instrumental support for caregiving is measured by whether children are reported as caregivers to older household members with functional limitations for Activities of daily living (ADL) or Instrumental activities of daily living (IADL) (Kim et al., 2024). ADL includes dressing, wearing footwear, walking across a room, bathing, eating, getting in or out of bed, and using the toilet. IADL includes preparing meals, grocery shopping, making telephone calls, taking medications, working around the house, managing money, and navigating outside the house. The caregiver is identified as the person whom the parents claim is the caregiver. We construct a binary indicator coded 1 if the member is listed as the caregiver and 0 otherwise. Caregiving in Indian households has a gendered aspect wherein, care is often provided by daughters-in-law (Ugargol and Bailey, 2018), even though property tends to remain within direct bloodlines. To account for this dynamic, we conduct our analysis at the couple level. Each married child-spouse pair is treated as the unit of analysis and is coded as a caregiver if either spouse is a caregiver.

#### 3.3.3 Other controls

As discussed earlier, property transfers in India may happen within the broader gendered patrilineal norms. To account for these norms, we control for children's sex (Jayachandran and Pande, 2017). Previous research suggests that birth order is associated with migration and patterns of asset ownership, both of which may shape living arrangements (Fernando, 2022). We therefore include birth order as a control variable. We do not have information about the life stage of all children, which can also influence their ownership likelihood (Le Goff et al., 2017). To proxy for this missing detail, we include the child's age as a control. We also control for each child's education, measured by years of schooling, as a proxy for income, following a similar approach to McGarry (1999).

#### 3.3.4 Analytical approach

To examine the association between adult children's residential proximity to their parents and ownership of immovable assets reported within the parental household's asset portfolio, we estimated a conditional fixed-effects logistic regression (conditional logit) model at the family level. The model exploits within-family variation across siblings, thereby controlling for all observed and unobserved family-level characteristics common to all siblings, including parental characteristics and other time-invariant parental household factors. Consequently, identification relies on within family comparisons among adult siblings in families where some siblings own parental household property, and others do not. The conditional family fixed-effect model is specified in Equation 1 below.


$$\begin{array}{l} P\left(O_{ik}=1|{\alpha_{k},D_{ik},\;X}_{ik}\right)=F\left(\alpha_{k}+\beta D_{ik}{+\;\gamma X}_{ik}\right) \end{array}$$ (1)

where *O*_*ik*_ is a binary indicator that equals 1 when the *i*_*th*_ child of the *k*_*th*_ parent is an owner (=1) and 0 otherwise; α_*k*_ denotes the family-specific fixed effects; *D*_*ik*_ represents the indicator of child's proximity to the parental household; and *X*_*ik*_ is a vector of the child-level control variables discussed earlier. The function *F(.)* denotes the cumulative logistic distribution function, specified in Equation 2 below:


$$\begin{array}{l} F\left(z\right)=\frac{\exp\left(z\right)}{1+\exp\left(z\right)} \end{array}$$ (2)

Although the specification includes a family-specific fixed-effect α_*k*_, estimation is done using the conditional likelihood, which conditions out this parameter (Chamberlain, 1980). Marginal effects following conditional (household fixed-effects) logistic regression are reported as average marginal effects (AMEs) for ease of interpretation. The AMEs summarize the average change in the predicted probability of ownership associated with a change in a covariate, holding other variables at their observed value.^5^ Robust standard errors are clustered at the family level, and the standard errors of the estimated AMEs are obtained using the delta method. As a complementary check, we estimated linear probability models with household fixed effects using both the full analytic sample and the conditional logit estimation sample. All regressions were weighted using the harmonized LASI household probability weights.

Finally, we examine heterogeneity in the association by household wealth, the educational attainment of the household head, the regional zone, caste affiliation, and parent age group.

## 4 Results

### 4.1 Descriptive analysis

Table 2 presents descriptive statistics for the sample of children used for the analysis. The descriptive statistics are presented separately for the full estimation sample of children, co-resident children, children residing in the same city or village, and children residing outside the city or village. Our full sample comprises 9,124 children from 2,365 parental households. In our full sample of children, 36.28% are reported as the property owners of at least one of the properties within the household's portfolio. However, among co-resident children, the ownership proportion increases to 57.87%, suggesting a possible link between co-residence and ownership. On the other hand, only 19.52% of children who do not reside in the household but are in the same city or village are listed as owners. The proportion increases slightly for those outside the city or village to 24.04%, but remains significantly lower than for co-resident children.

**Table 2:** Descriptive characteristics of the children in the estimation sample.

Variables	Total	Co-resident children	Non-resident children within the same city or village	Non-resident children outside the city or village
	Mean (SD)/%	Mean (SD)/%	Mean (SD)/%	Mean (SD)/%
Property owners, %	36.28	57.87	19.52	24.04
Single owners, %	21.85	27.72	16.12	19.79
Joint owners, %	15.75	32.92	3.50	4.85
Own current residence, %	23.58	35.09	14.11	17.62
Own other property, %	6.74	9.92	2.90	6.42
Own cultivable land, %	13.73	21.42	7.67	9.48
Own non-cultivable land, %	4.76	4.68	3.18	6.57
Age of child, in years	36.02 (10.23)	33.01 (10.02)	38.76 (9.61)	37.28 (10.09)
Sex (Ref. Male)				
Female, %	46.26	20.01	53.29	75.58
Years of schooling	8.40 (5.13)	9.83 (4.58)	7.00 (5.07)	7.92 (5.41)
Child birth order, %				
1 (Oldest)	26.33	22.94	29.03	28.16
2	26.46	27.48	25.74	25.79
3	20.59	21.12	19.73	20.79
4 and above	26.62	28.46	25.5	25.26
Caregivers, %	2.96	7.09	0.25	0.08
Children	9,124	3,679	2,828	2,617

SD, standard deviation of the variable. A child is classified as a sole owner if they are the exclusive owner of at least one of the following immovable assets reported in the parental household: the current residence, other residential or non-residential property, cultivable land, or non-cultivable land. Similarly, a child is classified as a joint owner if they jointly own at least one of these asset types. These categories are not mutually exclusive; a child may be the sole owner of one asset and a joint owner of another. Consequently, the proportions of sole and joint owners do not sum to the overall proportion of owners, and the proportions reported for individual asset types do not sum to the overall proportion of property owners because a child may own more than one type of immovable asset.

Sole ownership is more common than joint ownership in the full sample. However, co-resident children are more likely to be joint owners while non-resident children are more likely to be single owners of the property. Note that sole ownership refers to exclusive ownership of at least one reported immovable asset, whereas joint ownership refers to ownership of at least one reported immovable asset jointly with another family member. These categories are not mutually exclusive, as a child may be the sole owner of one asset and a joint owner of another. In our sample, however, such overlap is limited. Only 121 children are classified as both sole and joint owners (approximately 6% of sole owners and 8% of joint owners).

The average age of children in our sample is 36 years, but co-resident children are younger. As unmarried and younger children often co-reside with parents unless they migrate for employment or marriage, the age pattern is expected. Nearly 46% of the children are daughters. However, consistent with patrilocal residence patterns, daughters make up only 20% of the co-resident children (Goli et al., 2024). Among the children residing outside the city or village, they make up 75% of all children. The observed ownership differences may also reflect gendered inheritance norms. In the patrilineal inheritance norms prevalent in the country, daughters are generally less likely to be a beneficiary of the parental property (Deininger et al., 2013). In our subsequent analysis, we control for the child's gender and evaluate our results for the restricted sample of sons to isolate the co-residence effect more clearly.

Co-resident children have received more years of schooling compared to others. This may mean their earning capacity is higher and, hence, their ability to purchase an asset as part of the household portfolio. We assess the possibility of children's financial contribution to the asset purchase in our robustness analysis. We also measure the caregiving at the intensive and extensive margins for these children. In our sample, 2.96% children or their spouses are caregivers; almost all of these caregivers are co-resident children who provide care over nearly 20 days a month. This is expected as co-resident children are best positioned to provide care. This pattern is consistent with the possibility that caregiving can contribute to the observed association between co-residence and ownership. We examine the association formally in the subsequent analysis.

The sample households come from all six zones of India (Appendix Table A1).^6^ Approximately 30% of the households belong to Scheduled Castes or Scheduled Tribes, 37% to Other Backward Classes, and 32% to other/no caste groups. Considering regional and caste-based variation in kinship (Karve, 1968), we examine heterogeneity in our results by caste and zone in the heterogeneity section. Compared with the analytic sample, households included in the estimation sample tend to be larger, have more adult children, and are wealthier. These differences reflect the estimation requirements of the conditional fixed-effects logit model, which relies only on households contributing to the conditional likelihood. Accordingly, the estimation sample should be interpreted as a selected subset of the analytic sample rather than as fully representative of all households.

### 4.2 Do co-resident children hold greater ownership of household property?

In Table 3, we present the average marginal effects from a family fixed-effects conditional logit model that estimates the relationship between the child's proximity and their likelihood of ownership of immovable assets reported within the parental household's asset portfolio. Relative to co-resident children, those living in the same city or village but outside the parental household are associated with a 30 percentage-point lower probability of being reported as owners of the parental household's immovable assets, while children residing outside the city or village are associated with a 20 percentage-point lower probability, as shown in Column I. The results are significant at 1% level. The results therefore support the first hypothesis, which states that co-residing with a parent is associated with a higher likelihood of parental household asset ownership. These patterns are consistent with an exchange-based interpretation, although alternative explanations, including employment opportunities, housing constraints, economic considerations, and family preferences, cannot be ruled out.

**Table 3:** Regression results examining association between the proximity of the child and the likelihood of owning property in parental household.

Variables	DV: Owns any immovable property	DV: Owns any immovable property	DV: Owns any immovable property	DV: Joint owner of any immovable property	DV: Sole owner of any immovable property
	Conditional FE logit	LPM (Full analytic sample)	LPM (Estimation sample)	Conditional FE logit	Conditional FE logit
	(I)	(II)	(III)	(IV)	(V)
Proximity (Ref. Co-residence)					
Within the city/village	−0.30^***^	−0.05^***^	−0.43^***^	−0.43^**^	−0.17^***^
	(0.07)	(0.00)	(0.03)	(0.21)	(0.06)
Outside the city/village	−0.20^***^	−0.05^***^	−0.34^***^	−0.32^*^	−0.07^*^
	(0.06)	(0.00)	(0.04)	(0.18)	(0.04)
Years of schooling	0.00	0.00^*^	0.00	0.00	0.00
	(0.00)	(0.00)	(0.00)	(0.00)	(0.00)
Age of child (in years)	0.01^***^	0.00^***^	0.01^***^	0.01^***^	0.01^***^
	(0.00)	(0.00)	(0.00)	(0.00)	(0.00)
Daughter (Ref. Son)	−0.11^***^	−0.01^***^	−0.12^***^	−0.13^***^	−0.07^**^
	(0.03)	(0.00)	(0.02)	(0.04)	(0.03)
Birth order	0.00	0.00	0.00	0.01	0.00
	(0.01)	(0.00)	(0.01)	(0.01)	(0.01)
Constant		0.00	0.23		
		(0.02)	(0.15)		
Households	2,365	33,198	2,365	1,057	1,443
Observations	9,124	1,05,094	9,124	4,059	5,558

^*^p < 0.1, ^**^p < 0.05, ^***^p < 0.01.

DV denotes the dependent variable in the corresponding regression; FE refers to Fixed Effects and LPM refers to Linear Probability Model. Columns I, IV, V report average marginal effects (AMEs) computed after estimating conditional fixed-effects logit models. Columns II, III report coefficients from linear probability models (LPMs), estimated using the full analytic sample and the estimation sample corresponding to the conditional fixed-effects logit model, respectively. For Columns I, IV, V, standard errors are computed using the delta method and clustered at the household level. For Columns II, III robust standard errors clustered at the household level are reported. Property refers to any immovable asset reported within the parental household, including the current residence, other residential or non-residential property, cultivable land, and non-cultivable land. All regressions are estimated using household sampling weights.

In line with the country's cultural landscape, sons are more likely to own household assets, as evidenced by the significant, negative association with being a female child. Older children are also more likely to be the owners. We do not find evidence that ownership arrangements differ systematically between first-born and later-born children. We also observe that more educated children are only marginally more likely to be the owners.

Columns II and III of Table 3 report linear probability models estimated on the full analytic sample and on the conditional fixed-effects estimation sample, respectively. The probability of a sibling being an owner of the parental household property is lower by 5% if they are not co-residing and living in another household within the city/village, as per the full sample. In the restricted sample (same as conditional logit), the effect is 43%. Hence, our LPM results align with those of the conditional logit model.

We also estimate separate models for joint and single ownership in Columns IV and V of Table 3. Shared ownership allows retention of control rights over the property (Besley and Ghatak, 2010). In the context of intergenerational transfers, such arrangements of ownership may also preserve parents' bargaining position to secure future care. The estimated association between residential proximity and ownership is larger for joint ownership than for sole ownership, consistent with the earlier premise, although both associations remain statistically significant. The sole- and joint-ownership outcomes are not mutually exclusive, as a child may be the sole owner of one asset and a joint owner of another. However, as there is limited overlap in our sample, the two models largely capture distinct ownership arrangements.

### 4.3 Exploring the role of caregiving

We examine whether observed caregiving partly explains the association between residential proximity and property ownership. We do this by restricting the analysis to households in which at least one adult reports a limitation with ADL or IADL and assessing whether the association between co-residence and ownership attenuates upon controlling for caregiving provision as per the second hypothesis. The results are presented in Table 4.

**Table 4:** Regression results examining the role of caregiving.

Sample	DV: Owns any immovable property	DV: Owns any immovable property	DV: Owns any immovable property
	All children in households with care needs^a^	All children in households with care needs	Co-resident children in households with care needs
	(I)	(II)	(III)
Proximity (Ref. Co-residence)			
Within the city/village	−0.25^**^	−0.20^**^	
	(0.11)	(0.10)	
Outside the city/village	−0.18^**^	−0.14^*^	
	(0.09)	(0.08)	
Is child a caregiver?		0.10^*^	0.04
		(0.05)	(0.04)
Sex (Ref. Male)			
Female	−0.10^**^	−0.09^*^	−0.15
	(0.04)	(0.05)	(0.14)
Age of member	0.01^***^	0.01^***^	0.01^**^
	(0.00)	(0.00)	(0.00)
Years of schooling	0.00	0.00	0.00
	(0.00)	(0.00)	(0.00)
Birth order	0.01	0.01	−0.01
	(0.01)	(0.01)	(0.02)
Households	1,284	1,284	274
Observations	5,077	5,077	706

^*^p < 0.1, ^**^p < 0.05, ^***^p < 0.01.

DV denotes the dependent variable in the corresponding regression. Average marginal effects (AMEs) are reported for all regressions. Robust standard errors, computed using the delta method and clustered at the household level, are reported in parentheses. Property refers to any immovable property owned by the household, including the current residence, other residential property, cultivable land, and non-cultivable land. All regressions are estimated using household sampling weights.

^a^Households with care needs refers to those households where as at least one adult reports a limitation with any one Activities of daily living or Instrumental activities of daily living"

Column I of Table 4 presents estimates for children of the household heads in the households where any older adult requires care with ADL or IADL. In these households, children who do not co-reside but live in the same village or city are associated with a 25 percentage-point lower probability of being reported as owners of the household's immovable assets relative to co-resident children. After controlling for whether the child provides care to any adult, this gap narrows to 20 percentage points, as shown in Column II, suggesting only a partial attenuation of the proximity effect. A similar drop can also be seen in the likelihood of ownership among children residing outside the city or village. The modest attenuation provides only limited support for the proposition that observed caregiving explains the association between co-residence and ownership.

Finally, in Column III, we examine whether caregiving is associated with a further ownership advantage among co-residing children. Among co-resident children, caregivers are 4 percentage points more likely to be property owners, although the estimate is not statistically significant. This suggests that, conditional on co-residence, there is limited additional variation in ownership explained by observed ADL/IADL caregiving. One possible explanation is that in joint households, caregiving responsibilities are often informally shared among siblings or differentiated across domains (e.g., direct care vs. financial or administrative support), making observed ADL/IADL caregiving an incomplete measure of the support exchanged within families (Ikkink et al., 1999; Jothikaran et al., 2023). It is also possible that the association between co-residence and ownership may reflect expected future care, children's availability, preferences, administrative help, or broader filial norms that are not captured by the observed caregiving measures.

### 4.4 Robustness checks

We conduct multiple robustness checks of our main results. The first concerns the sample of children that we use for the analysis. Under patrilocal norms, daughters typically reside with their husband's family, and as per patrilineal customs, property is customarily transferred to sons. Including non-resident daughters in the sample may, therefore, confound the observed effect, as sons will both co-reside and be more likely to be property owners, in accordance with cultural norms. To avoid this confounding, we restrict the sample to sons only in Table 5, Column I. We find that co-resident sons are now 46 percentage points more likely than non-resident sons within the same city or village to own parental household assets at the 1% significance level. Sons who are living outside the city or village are also 37 percentage points less likely to be property owners than co-resident sons. These results suggest that the main association is not driven solely by comparisons between co-resident sons and non-resident daughters, but also holds among sons who differ in their residential proximity to parents.

**Table 5:** Robustness checks for the association between residential proximity and property ownership.

Variables	DV: Owns any immovable property	DV: Owns current residence	DV: Owns current residence	DV: Owns current residence	DV: Owns any immovable property
	Sons only	Restricted ownership definition	Restricted definition; Single non-resident child	Current residence acquired before child reached adulthood	Households without current ADL/IADL care needs^a^
	(I)	(II)	(III)	(IV)	(V)
Proximity (Ref. Co-residence)					
Within the city/village	−0.46^***^	−0.42^**^	−0.44	−0.28^***^	−0.36^***^
	(0.04)	(0.20)	(0.37)	(0.06)	(0.08)
Outside the city/village	−0.37^***^	−0.34^**^	−0.28^**^	−0.03	−0.22^***^
	(0.07)	(0.13)	(0.14)	(0.04)	(0.08)
Years of schooling	0.00	0.00	0.01	0.00	0.00
	(0.00)	(0.00)	(0.01)	(0.00)	(0.00)
Age of child	0.01^*^	0.00	0.00	0.01^*^	0.01^***^
	(0.00)	(0.00)	(0.01)	(0.00)	(0.00)
Daughter (Ref. Son)		−0.10^***^	−0.06	−0.10^**^	−0.11^***^
		(0.03)	(0.05)	(0.04)	(0.03)
Birth order	−0.03	−0.02^**^	−0.10^***^	−0.01	−0.01
	(0.02)	(0.01)	(0.02)	(0.02)	(0.02)
Households	1,265	1,304	361	843	1,081
Observations	3,519	4,972	897	3,037	4,047

^*^p < 0.1, ^**^p < 0.05, ^***^p < 0.01.

DV denotes the dependent variable in the corresponding regression. Average marginal effects (AMEs) are reported. Robust standard errors, computed using the delta method and clustered at the household level, are reported in parentheses. Column II uses a more conservative definition of property ownership for non-resident children, whereby only those explicitly identified as child owners are classified as owners. Column III further restricts the sample to households with only one non-resident child, thereby minimizing ambiguity in assigning ownership. Property refers to any immovable asset owned by the household, including the current residence, other residential property, cultivable land, and non-cultivable land. All regressions are estimated using household sampling weights.

^a^No older members of the household need help with an activity of daily living (ADL) or an instrumental activity of daily living (IADL).

Furthermore, because ownership of non-resident children is not always explicitly identified in LASI, our main specification attributes ownership to a non-resident child when the recorded owner is a non-household member. As a robustness check, we adopt a more restrictive ownership definition that classifies a non-household child as an owner only when the non-household owner is explicitly identified as a child. Our results, shown in Column II of Table 5, align with the main findings. We further refine the sample by restricting to only those parental households with only one child outside the household, to minimize ambiguity about which non-resident child is the recorded owner. The estimated associations shown in Column III of Table 5 remain directionally consistent with the main specification, although the standard errors increase likely because of the smaller sample.

One potential concern in the analysis is that we do not distinguish between ownership reflecting transfers from parents and ownership reflecting property purchased by children themselves. For the current residence, we know the year it was acquired. We therefore exploit this information to assess the potential financial contribution children might have made toward property purchases. This restriction makes it less likely that children contributed financially to the original purchase of the parental residential property. Accordingly, we restrict our sample to families in which the house was acquired before the child had turned eighteen.^7^ Our results in Column IV of Table 5 indicate that co-resident children are 28 percentage points more likely to own parental residential property than non-resident children within the same city or village. However, there is no significant difference between co-resident children and non-resident children outside the city under this specification. The results therefore, reduce concerns that the observed association is driven by children's financial contribution to the purchase. However, it does not identify when ownership was transferred, and thus does not resolve the timing-of-transfer limitation.

Finally, an alternative explanation for the observed association is that co-residence reflects contemporaneous caregiving needs rather than broader intergenerational arrangements. To examine this possibility, we conduct a robustness check by restricting the sample to households in which no older adult reports any ADL/IADL care needs. Such a restriction allows us to examine whether the association between residential proximity and ownership persists in households where no current ADL/IADL care needs are reported. The results are shown in Column V of Table 5. Co-resident children have a 36 percentage-point higher probability of ownership than children living in the same city or village but in a separate household. This suggests that the observed association is not solely driven by households with current ADL/IADL care needs.

### 4.5 Heterogeneity results

**Figure 1:**
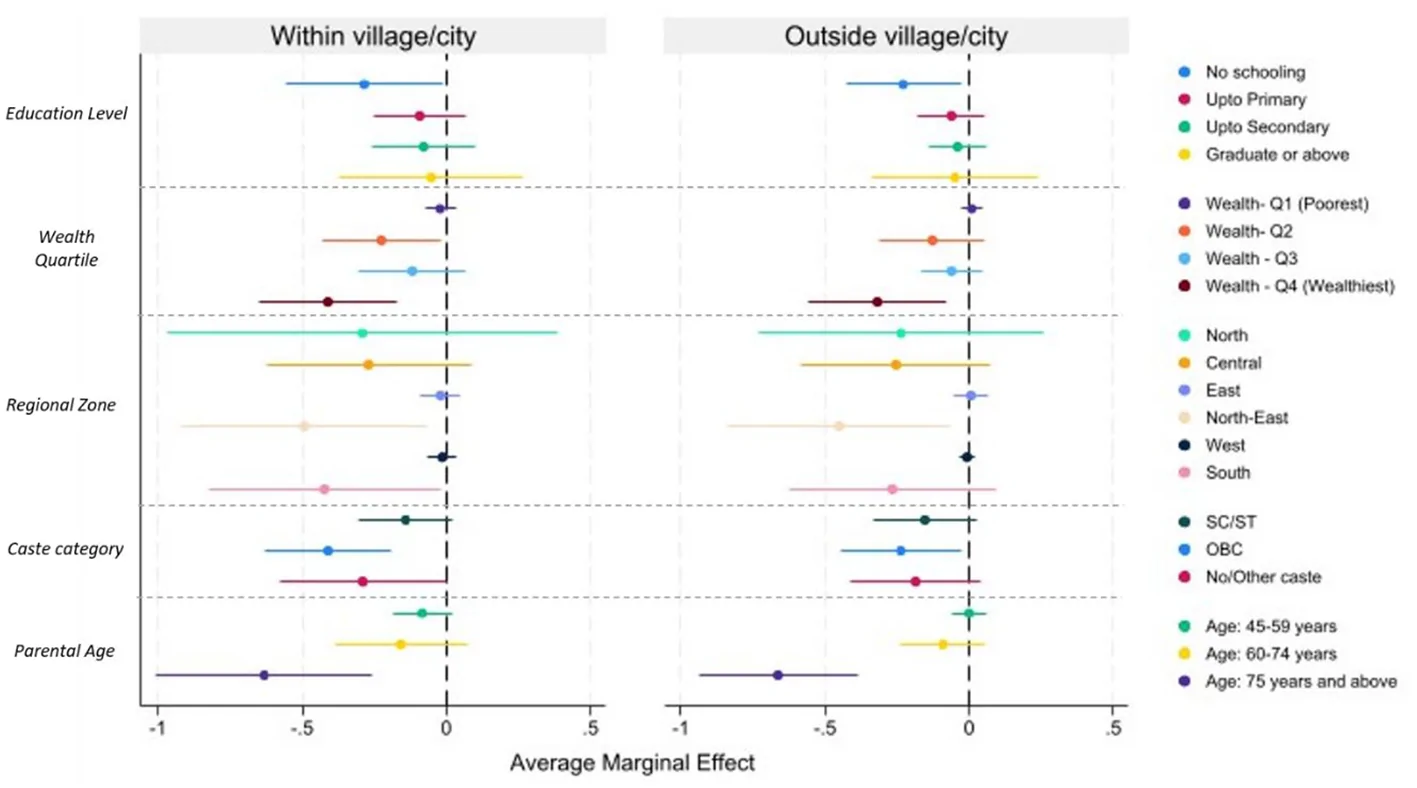
Heterogeneity results of the association between proximity and property ownership across subgroups. Markers represent subgroup-specific average marginal effects (AMEs) estimated from family fixed-effects conditional logit models of property ownership on residential proximity. Error bars indicate 95% confidence intervals.

Figure 1 presents the subgroup estimates examining whether the association between residential proximity and ownership varies across key household and social characteristics. To formally assess whether subgroup differences are statistically meaningful, we estimate interaction models and report Wald tests of the interaction terms in Appendix Table A2. Detailed regression estimates are reported in Appendix Table A3.

Educational attainment may be associated with differences in property ownership arrangements. We therefore examine whether the association between residential proximity and ownership differs by the parents' educational attainment.^8^ We estimate separate models for four education groups: no schooling, up to primary, up to secondary, and graduate or above. Among households headed by individuals with lower educational attainment, co-residence is associated with a larger, statistically significant increase in children's ownership compared with children living elsewhere within the same city or village. The corresponding estimates are smaller among households headed by individuals with upper secondary or tertiary education. However, Wald tests of interaction indicate that the differences across education groups are not statistically significant (Appendix Table A2).

Total parental household wealth may also be associated with differences in ownership arrangements. Households with greater asset holdings may have greater flexibility in allocating ownership among children. To examine this possibility, we partition households into quartiles based on total household net asset value and estimate separate models for each quartile. The estimates in Figure 1 indicate that the negative association between non-co-residence and ownership is strongest in the wealthiest quartile. Interaction tests indicate that these differences across wealth groups are statistically significant (Appendix Table A2), suggesting that household wealth plays a role in the relationship.

Furthermore, as kinship systems, inheritance practices, and family organization differ across regions and caste groups, we examine whether the association between residential proximity and ownership varies across these social contexts. Figure 1 presents separate estimates for each regional zone. Across all regions, co-resident children are more likely to be reported as owners than non-resident children, although several estimates are imprecisely estimated. We also estimate separate models by caste category. The association is observed across all caste groups, although the estimated magnitude is larger among Other Backward Classes (OBCs) and households classified as Other/No Caste. Wald interaction tests indicate statistically significant differences across regional or caste groups (Appendix Table A2).

Next, we check heterogeneity by parental age group. As parents age and experience declining health or functional limitations, the value of support that co-resident children can provide increases (Ugargol et al., 2016). Under exchange theory, this may increase the salience of reciprocal exchange arrangements. Our results shown in Figure 1, indicate that the association between co-residence and ownership is indeed stronger among older parents, consistent with an exchange-based interpretation. Interaction tests confirm that these differences are statistically significant (Appendix Table A2). We also examine heterogeneity by urban/rural residence and number of rooms, as these characteristics may reflect differences in residential context and housing conditions, but interaction tests indicate no statistically significant differences (Appendix Table A2).

Taken together, these findings suggest that the association between residential proximity and property ownership exhibits heterogeneity across socioeconomic groups.

## 5 Discussion

In this study, we examined whether adult children who co-reside with their parents are more likely to own parental household property than their siblings who do not co-reside, a pattern consistent with reciprocal intergenerational support arrangements. Unlike most other studies that analyze reciprocity through financial transfers or bequests, we leverage the ownership structure of immovable household assets to assess whether the observed ownership patterns of parental household assets are consistent with the exchange-based interpretation of intergenerational support. We are among the few studies to empirically examine such ownership arrangements in the Indian context.

Using within-family variation in ownership among adult children of the household head, our conditional fixed-effects logit estimates reveal a systematic association between property ownership and co-residence, with co-resident children being more likely to own parental household property than their siblings residing elsewhere. The association is stronger when comparing co-resident children with siblings living in a separate household within the same city or village than when comparing them with siblings living outside the city or village. These findings are robust to a range of alternative specifications. We also find that caregiving for ADL/IADL accounts for only a small part of this association, suggesting that other factors may also contribute to this relationship.

The findings of our study are broadly consistent with exchange theory, which postulates a quid pro quo arrangement between parents and children (Bernheim et al., 1985; Brown, 2006; Fu, 2019; Groneck, 2017). This theory suggests that parents may use financial incentives to influence their children's behavior. Consistent with this perspective, co-resident children may be more likely to hold property ownership because residential proximity facilitates intergenerational support and caregiving arrangements (Bengtson and Roberts, 1991). However, since we observe only property ownership rather than the motives or the timing of ownership transfers, our results should be interpreted as suggestive rather than as causal evidence of an exchange motive. The findings, furthermore, do not rule out alternative explanations for the observed association that cannot be distinguished using the available data.

The study contributes to family and aging research by highlighting that property ownership may be embedded within intergenerational support arrangements in contexts where family remains the dominant source of old-age security. The findings are particularly relevant in the Indian context, where families have traditionally been the primary providers of support for aging members (Karve, 1968; Kumar, 1995; Uberoi, 1994). Underpinning this arrangement are social norms, moral obligation, altruistic motivation, and reciprocal dynamics. Informal family support remains the primary safety net for older adults in the country, as reflected in intergenerational co-residence and shared living arrangements (Bengtson and Roberts, 1991; NSO, 2021; Sathyanarayana et al., 2014). This assumes particular importance as formal social security arrangements are relatively underdeveloped (OECD, 2023). However, the traditional joint household structure is under strain due to urbanization, the migration of the younger generation from rural to urban areas, and industrialization (Allendorf, 2020; Bloom et al., 2010; Raju, 2014). Additionally, a large share of the household wealth is held in immovable assets, which are generally less liquid and therefore not readily accessible. In such a context, our findings suggest that immovable property may occupy a central place in intergenerational support arrangements. In doing so, the study extends a literature that has hitherto largely focused on intergenerational financial transfers (Cox, 1987; Cox and Rank, 1992) and bequests (Bernheim et al., 1985; Groneck, 2017) by highlighting the role of property ownership arrangements in intergenerational support.

The findings also underscore the importance of cultural factors in property allocation. We observe that sons are more likely to be property owners, in line with the country's patrilineal tradition. Furthermore, birth-order effects are apparent, as older children are more likely to be the owners. This indicates that family hierarchies also play an important role in shaping ownership decisions. We also find that the relationship between co-residence and asset ownership is stronger among wealthier households and older parents, suggesting a moderating effect of wealth and age. We further observe variation across regional and caste groups, suggesting that differences in social organization, kinship practices, and access to assets may shape the relationship between co-residence and property ownership.

Several alternative explanations remain possible. Co-residence may reflect filial obligations, preferences for joint living or cultural preferences (Karve, 1968; Uberoi, 1994), altruistic motivations (Becker, 1974), economic dependency of the child, housing constraints (Esteve and Reher, 2021), job location and migration (Fernando, 2022), or marital circumstances (Mishra and Kaur, 2021), rather than anticipated caregiving or reciprocal exchange alone. Co-residence may also reflect material constraints—housing prices, affordability, space constraints in the parental house can also influence decisions regarding co-residence (Esteve and Reher, 2021). These unobserved factors may be especially relevant for children living in the same city/village as the parent but not co-residing. While our conditional fixed-effects logit model removes shared parent-level characteristics, we observe only limited child-level characteristics in the data and therefore cannot control for all variables in our analysis. Similarly, property ownership may be shaped by inheritance practices or other family-specific considerations that are not directly observed in the data (Agarwal, 1995; Karve, 1968). Consequently, the findings should be interpreted as associational rather than causal.

Another limitation is that we cannot observe the timing of transfer. It is plausible that the transfer preceded the co-residence. Panel data on ownership can help precisely determine the timing of the transfer, which our cross-sectional data cannot. It is also possible that children who own property themselves may be more likely to host or support parents, rather than ownership being granted as an incentive for co-residence or care.

Furthermore, our primary ownership measure requires an assumption regarding the identity of the non-household owner. Although robustness analyses using more conservative ownership definitions yielded estimates in the same direction, we acknowledge the need for richer data that explicitly identifies ownership at the individual child level.

We also acknowledge that using a conditional fixed-effects model limits the generalizability of our findings, as estimation relies only on households that contribute to the conditional likelihood. Consequently, households with no within-family variation in property ownership do not contribute to estimation, resulting in a substantially smaller final estimation sample than the analytic sample. The estimation sample is larger and wealthier than the full analytical sample because the conditional fixed-effects logit model includes only households with within-family variation in the outcome. Accordingly, the findings should be interpreted as applying to this selected sample.

Identifying the motives underlying property ownership arrangements remains challenging. We acknowledge the need for more specific elicitation of the motives. It is also essential to analyze whether the inter vivos ownership arrangements we study ultimately translate into support and care in later life [a concern echoed by Chakrabarti (1995) and Rajan et al. (1999)]. Financial planning for old age has a long gestation period, and changing social and economic dynamics can alter the terms of the intergenerational contract within a family (Croll, 2006; Self, 2013). Future research using longitudinal data on ownership transfers and caregiving could help identify the timing and motivations underlying property arrangements and assess whether such arrangements ultimately translate into support in later life.

## 6 Conclusion

This study examines the association between co-residence and property ownership within families in India. By exploiting sibling-level differences in asset ownership using a nationally representative dataset, we provide suggestive evidence that ownership of parental household immovable assets is associated with co-residential arrangements in a subset of households, consistent with reciprocal intergenerational support arrangements. In doing so, the study contributes to the literature on living arrangements and reciprocal exchange within Indian families.

At the same time, these findings should be interpreted as reflecting descriptive patterns rather than causal relationships. Nevertheless, the findings suggest that property ownership may play a role in how some families organize intergenerational support and old-age security. Understanding these arrangements may therefore be important to understand how older adults secure support in contexts where family remains the primary source of care and household wealth is concentrated in illiquid assets such as land and housing.

## Data Availability

Publicly available datasets were analyzed in this study. This data can be found here: Gateway to Global Aging data at https://doi.org/10.25549/h-lasi. Raw data is also available upon request from International Institute for Population Sciences (IIPS) (iipsdata.ac.in).

## Footnotes

1 The adult children of the household head are referred to as siblings in the paper.

2 Inter vivos refers to gifts or transfers made during one's lifetime.

3 Note that the estimation sample includes all linked adult children, regardless of whether they reside in the parental household or not.

4 For ease of readability, we henceforth refer to non-co-resident child as non-resident child.

5 Because the family-specific fixed effects are conditioned out and not estimated in a conditional logit model, predicted probabilities as AMEs are evaluated with the fixed effect set to zero, following the implementation of Stata's margins command after clogit.

6 The regional classification follows the government's creation of zonal councils using each group of states. The classification is available at: Ministry of Home Affairs | Government of India.

7 As per The Majority Act, 1875, every Indian attains the age of majority on completing the age of 18 years.

8 Educational attainment is measured using the household head's education. When the household head was not interviewed, we instead use the educational attainment of the head's spouse.
