# Supplementary material for: Living arrangements and property ownership among families in India
Source: Front Sociol. 2026 Aug 25;11:1865674. doi: 10.3389/fsoc.2026.1865674 (PMC13549918; doi:10.3389/fsoc.2026.1865674)
Supplement: Supplementary file 1 [file Supplementary_file_1.docx]

**Appendix**

*Table A1: Descriptive comparison of the analytic and the final estimation sample*

| Variables | Analytic Sample | Final Estimation Sample |
| --- | --- | --- |
|  | Mean (SD)/ % | Mean (SD)/ % |
| Education of head/spouse, in years | 4.82 (4.94) | 4.09 (4.58) |
| Number of household members | 4.76 (2.57) | 5.85 (2.57) |
| Net household wealth, in INR | 2,598,017 (12,000,000) | 4,685,760 (18,100,000) |
| Number of living children | 3.79 (1.93) | 4.29 (1.94) |
| Age, in years | 60.48 (10.24) | 64.70 (10.69) |
| Rural, % | 64.59 | 68.33 |
| Age Group of parents |  |  |
| 45-59 years, % | 48.49 | 32.39 |
| 60-74 years, % | 41.21 | 48.88 |
| 75+ years, % | 10.30 | 18.73 |
| Zone |  |  |
| North, % | 16.06 | 19.41 |
| Central, % | 15.37 | 14.67 |
| East, % | 16.83 | 14.71 |
| North-East, % | 14.96 | 13.36 |
| West, % | 13.34 | 17.93 |
| South, % | 23.44 | 19.92 |
| Caste |  |  |
| SC/ST, % | 34.84 | 29.94 |
| OBC, % | 37.84 | 37.04 |
| No/Other caste, % | 26.56 | 32.01 |
| Households | 1,05,094 | 9,124 |
| Observations | 33,198 | 2,365 |

*Note*: The analytic sample includes all households that meet the study eligibility criteria and have complete information on the variables used in the analysis. The final estimation sample is smaller because conditional fixed-effects logit models are estimated using only households that contribute to the conditional likelihood. Consequently, households with no within-family variation in the dependent variable are excluded automatically from estimation.

*Table A2: Wald Test results from interaction analysis*

| Heterogenous Group | Joint Wald χ² | df | p-value |
| --- | --- | --- | --- |
| Education levels | 10.23 | 6 | 0.116 |
| Wealth levels | 47.34 | 6 | 0.000 |
| Zones | 90.35 | 10 | 0.000 |
| Caste Groups | 18.59 | 4 | 0.001 |
| Parental age-groups | 108.22 | 4 | 0.000 |
| Urban/Rural | 3.44 | 2 | 0.179 |
| # of rooms | 7.54 | 6 | 0.273 |

*Note*: Each row presents estimates from a separate conditional household fixed-effects logit model that includes an interaction term between residential proximity and the variable listed in that row. Wald tests assess the statistical significance of the interaction terms.

*Table A3: Heterogeneity results for all sub-group regressions*

| Sub-Group (Dependent Variable: Owns any immovable property) | Within the city/village (Ref. Co-resident) | Outside the city/village (Ref. Co-resident) | Households | Observations |
| --- | --- | --- | --- | --- |
|  | AME (SE) | AME (SE) | N | N |
| **Panel A. Wealth quartile** |  |  |  |  |
| Quartile I (Poorest) | −0.02 (0.03) | 0.01 (0.02) | 273 | 1,029 |
| Quartile II | −0.23** (0.11) | −0.13 (0.09) | 513 | 1,985 |
| Quartile III | −0.12 (0.10) | −0.06 (0.05) | 625 | 2,474 |
| Quartile IV (Wealthiest) | −0.41*** (0.12) | −0.32*** (0.12) | 953 | 3,633 |
| **Panel B. Education** |  |  |  |  |
| No schooling | −0.28** (0.14) | −0.23** (0.10) | 1,032 | 4,221 |
| Up to primary | −0.09 (0.08) | −0.06 (0.06) | 700 | 2,720 |
| Up to senior secondary | −0.08 (0.09) | −0.04 (0.05) | 546 | 1,912 |
| Graduate or above | −0.05 (0.16) | −0.05 (0.15) | 87 | 271 |
| **Panel C. Region** |  |  |  |  |
| North | −0.29 (0.34) | −0.24 (0.25) | 459 | 1,704 |
| Central | −0.27 (0.18) | −0.25 (0.17) | 347 | 1,472 |
| East | −0.02 (0.04) | 0.01 (0.03) | 348 | 1,387 |
| North-East | −0.49** (0.22) | −0.45** (0.20) | 316 | 1,352 |
| West | −0.01 (0.03) | −0.01 (0.01) | 424 | 1,553 |
| South | −0.42** (0.20) | −0.27 (0.18) | 471 | 1,656 |
| **Panel D. Social group** |  |  |  |  |
| SC/ST | −0.14* (0.08) | −0.15* (0.09) | 708 | 2,863 |
| OBC | −0.41*** (0.11) | −0.24** (0.11) | 876 | 3,366 |
| No/Other caste | −0.29* (0.15) | −0.19 (0.11) | 757 | 2,807 |
| **Panel E. Parental Age group** |  |  |  |  |
| 45 - 59 years | −0.08 (0.05) | 0.00 (0.03) | 766 | 2,492 |
| 60 - 74 years | −0.16 (0.12) | −0.09 (0.07) | 1,156 | 4,661 |
| 75 and more years | −0.63*** (0.19) | −0.66*** (0.14) | 443 | 1,971 |

* p< 0.10, ** p < 0.05, *** p < 0.01
*Note*: Average marginal effects (AMEs) are reported from family fixed-effects conditional logit models. Robust standard errors (SE), computed using the delta method and clustered at the household level, are reported in parentheses. The omitted category for proximity is co-residence. All models include the same set of controls as the main model. Estimates are reported separately for the subgroup indicated in each row. *Households* and *Observations* denote the number of households and child observations included in the corresponding subgroup estimation. Property may refer to any one of the immovable properties that the household owns, including the current residence, other properties, and land. All regressions are estimated using household sampling weights.
